# Study to Evaluate the Comparative Efficacy of Medhya Rasayana (Pharmacological) Versus Nonpharmacological Interventions in Management of Gadget Addiction in Children: Protocol for Parallel, Triple-Arm, Randomized Clinical Trial

**DOI:** 10.2196/51833

**Published:** 2024-11-11

**Authors:** Prasad Yewale, Renu Rathi, Swapnali Mate

**Affiliations:** 1 Mahatma Gandhi Ayurved College Hospital and Research Centre Datta Meghe Institute of Higher Education and Research (Deemed to be University) Sawangi Meghe Wardha India; 2 Dr Rajesh Kambe Ayurved College and Hospital Turkhed Murtijapur Maharshtra India

**Keywords:** addiction, Ayurveda, gadget addiction, children, Kaumarbhritya, Medhya Rasayana, yoga, complementary and alternative medicine

## Abstract

**Background:**

Gadget addiction is a common behavioral problem among children. It is known to hamper social and academic life as well as adversely affect the lives of children. Ayurveda offers many therapeutic modalities and Ayurvedic medicines that can be used in the management of gadget addiction in children. The purpose of this study is to evaluate and compare the effectiveness of nonpharmacological therapies and the pharmaceutical intervention Medhya Rasayana in treating childhood gadget addiction.

**Objective:**

This study aims to provide a detailed description of the study methodology that will be used to compare the efficacy of nonpharmacological versus pharmaceutical interventions in the treatment of children’s gadget addiction.

**Methods:**

A randomized, parallel, triple-arm interventional study will be conducted on diagnosed participants of gadget addiction with an age group of 6- to 16-year-old children, which will be selected and equally distributed in 2 groups. Group P will be given Medhya Rasayana (pharmacological intervention), group N will be nonpharmacological Ayurveda intervention, and group C (cognitive behavioral therapy) will be an external group. The Study duration is 180 days with assessment at baseline, midpoint, and endpoint Appropriate statistical techniques, such as ANOVA and regression analysis, will be used to examine the data and evaluate the efficacy of the 3 groups’ interventions. We will perform subgroup analysis according to initial addiction severity, gender, and age. Primary outcome measures include a reduction in gadget addiction and changes in the psychosocial well-being of participants. Standardized questionnaires and instruments will be used to collect data.

**Results:**

In December 2023, the randomized controlled study got underway. Since participants may begin at any time, our goal is for everyone to be finished by December 2024.

**Conclusions:**

This research will provide crucial new information about the relative effectiveness of Ayurveda nonpharmacological therapies and Medhya Rasayana in treating children’s gadget addiction. The results will guide evidence-based treatments aimed at reducing the negative impact of excessive gadget use on this susceptible population’s psychosocial development. In the end, the findings are meant to help policy makers and medical professionals create sensible plans to deal with the rising issue of childhood gadget addiction.

**International Registered Report Identifier (IRRID):**

PRR1-10.2196/51833

## Introduction

### Overview

In the modern era, technology has affected life in various ways. One of the evolutions in the world of technology is electronic gadgets, which are used for entertainment, communication, information, education, and business. Moderate use of electronic gadgets improves the quality of life, but they also have the potential to be misused and cause gadget addiction. In general, addiction terminology was only associated with addiction to drugs, alcohol, and tobacco, but in the recent era, addiction may be defined as a process whereby a behavior that can function both to produce pleasure and to provide relief from internal discomfort is used in a pattern characterized by recurrent failure to control the behavior and continuation of the behavior despite significant negative consequences (unmanageability) [1]. Gadget addiction is a broad term that includes excessive use of mobile devices, internet games, internet-based gambling, excessive surfing on the internet, and excessive use of social media [2]. Gadget addiction is adversely associated with psychosocial disorders among children in the adolescent age group [3], as well as musculoskeletal problems and visual disturbance [4]. Gadget addiction leads to a reduction in physical activities, which results in fat deposition at an early age [5]. Research reveals that stressful environmental conditions promote human addiction [6]. Gadget addiction is a type of behavioral addiction; these addictions are similar to drug addiction except that in the case of gadget addiction, the person is not addicted to the substance but to the behavior or feeling brought about by the use of a gadget [7]. The mesolimbic brain circuit plays a significant role in behavior due to addiction [8]. All addictive substances increase the feeling of joy and this trend leads to their repeated use.

Previous studies by Ayurveda scholars reveal only the prevalence and side effects of gadget addiction but it lacking in proven and published cost-effective, promising, and convenient Ayurveda therapy for gadget-addicted children. Are search gap exists with special reference to the use of Ayurvedic interventions for the management of gadget addiction in children.

The Ayurveda concepts of Asatmeindriyartha Samyoga [9] (inadequate association of senses with their organs of perception) and Pradnyaparadh (misuse of intellect) go much further to today’s gadget addiction. Rather, it helps stimulate gadget addiction. The word Pradnyaparadha [10] is a combination of 2 words, that are Pradnya and Aparadh. Pradnya encompasses Dhee (intellect), Dhruti (control), and Smriti (memory), and Aparadh means misdemeanor. In addiction (could be of any type), the mental ability of the affected person is compromised, affecting their decision-making power. The mind needs controlling power to make decisions. According to Ayurveda, Dhee is the factor that governs the action of the mind [11]. Satva, Raja, and Tama Manas Guna also play an important role in the decision-making power and emotions of a person. Many features of gadget addiction are similar to the clinical features of Raja and Tama Guna’s predominance [12]. Hence, to achieve a normal mental equilibrium in a person, we need to increase satva guna and decrease Tama and Raja Guna. This can be achieved by adequate counseling, assurance, Sadvritta Palana (Ayurveda rules to prevent mental and physical disorders), Dhyana (meditation), and the use of the Medhya Rasayana [13]. Medhya Rasayana means the drugs that promote Medha, which has Dhee (intellect), Dhruti (control on mind), and Smriti (memory) as its components [14]. Medhya Rasayana enhances the Sadhaka pitta, which is responsible for Nischayatmaka Buddhi [15] that is the decision-making power to opt for the right thing over wrong.

Gadget addiction is a behavioral disorder. There is research evidence of the use of antidepressants and antianxiolytic drugs for the management of gadget addiction [16]. The antidepressant and anxiolytic effects of Medhya drugs have proven efficacy, as reflected in various previous studies [17]. Medhya Rasayana acts on the hypothalamus-pituitary-adrenal axis and normalizes the secretion of serotonin and acetylcholine, thereby improving mental function [18]. The classical texts of Ayurveda describe Satvavajaya Chikitsa (subjugation of mind) for the management and prevention of behavioral, psychogenic, and lifestyle-related disorders [19]. Satvavajaya (subjugation of mind) helps a person to control the mind and restrain it from unwholesome objects by playing a significant role in maintaining a harmonious state between intellect, memory, and patience, leading to a healthy mental condition. Practical approaches of Satvavajaya Chikitsa are Ashwasana (reassurance and explanation), Pratidwanda Chikitsa (replacement of emotions), Chintya (presumption), Dhyeya (correction of objectives and ideas), Sankalpa (proper guidance and advice for taking the right decision), Dhriti (proper control of patience), Suhritvakya (guidance and suggestion), Dharmarthavakya (education of individual and family), Santwana (rehabilitation and assurance) [20]. Applying the above methods could change children’s thinking and restrain them from unwholesome things. It could be beneficial in the management of Gadget addiction. Changing the thinking power of a patient is very important in the case of any addiction; this will happen with the help of Satvavajaya Chikitsa. Sadvritta helps in maintaining physical and mental fitness [21]. This helps improve the quality and longevity of life by secreting happy hormones [22]. It supports an increase in the Satvik guna of mind. Nonpharmacological measures like Sattwavajaya Chikitsa (subjugation of mind) dealing with manonigraha (control over unwanted thoughts and desires of mind) and proper counseling of patients, along with improvising the patient’s self-confidence. Along with this, yogic (postures and meditation) therapy and lifestyle modifications are very beneficial to keep the body and mind healthy and calm. Yoga enables the person to restrain from addiction by promoting self-regulation. Yoga has the potential to detoxify our mind and body and it is also capable of controlling emotions. This phenomenon is useful in addiction craving, compulsive behavior, tolerance, and relapse conditions [23]. Yoga induces dopamine homeostasis, leading to long-term benefits for various addictive behaviors [24].

### Protocol

#### Study Objectives

Evaluation of comparative efficacy of Medhya Rasayana (pharmacological) intervention versus Ayurveda nonpharmacological interventions and cognitive behavioral therapy in gadget addiction in children of the age group of 6 to 16 years.

The objectives of this study are listed in Textbox 1.

**Primary and secondary objective.**

**Primary objectives**

- To evaluate the efficacy of Medhya Rasayana in gadget addiction with an internet addiction test scale, a digital addiction scale, and *DSM-5* (*Diagnostic and Statistical Manual of Mental Disorders* [Fifth Edition]).
- To evaluate the efficacy of Ayurveda nonpharmacological interventions in gadget addiction with an Internet addiction test scale, a digital addiction scale, and *DSM-5*.
- To assess the comparative efficacy of 3 therapeutic modalities in the management of gadget addiction by Internet addiction test scale and *DSM-5*.

**Secondary objectives**

- To evaluate the association of gadget addiction with Deha and Manas Prakriti (physical and mental constitution) of the participant with the help of Ayusoft software. Manufactured by C-DAC (Centre for Development of Advanced Computing) To assess the impact of gadget addiction on social relationships and behaviors in the *DSM-5* scale.

#### Hypothesis

##### Null Hypothesis (H0)

Any intervention among the Medhya Rasayana and Ayurveda nonpharmacological intervention are not as effective as the control group in reducing gadget addiction in children.

##### Alternative Hypothesis (H1)

At least 1 intervention among the Medhya Rasayana and Ayurveda nonpharmacological intervention are more effective than the control group in reducing gadget addiction in children.

The purpose of this hypothesis is to determine whether the 3 interventions under comparison in the study differ in their efficacy. If statistically significant differences exist in the outcomes, researchers can ascertain them by testing the null hypothesis against the data gathered during the randomized controlled trial.

## Methods

### Study Design

**Figure 1:**
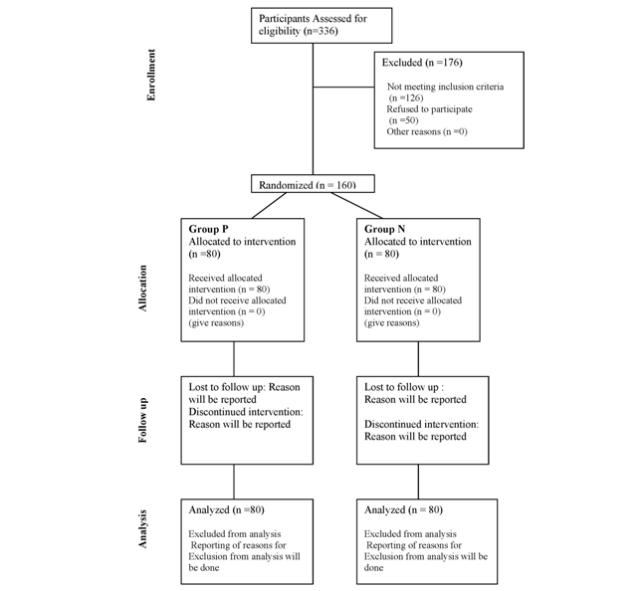
Consolidated Standards of Reporting Trials (CONSORT) flow diagram.

A 3-arm superiority randomized controlled trial will be performed. The study flow is depicted in Figure 1. Patients with gadget addiction will be selected from the Kaumarbhritya Outpatient Department of MGACHRC Salod, Wardha as per inclusion criteria mentioned in Textbox 2. During the first visit, Parents will be asked whether they would like to deaddict their child from gadget addiction if they are willing then written informed assent will be obtained from the participants’ guardian. Participants will then complete questionnaires on the internet addiction test scale and digital addiction scale. Furthermore, participants’ social behavior and Prakruti (physical and mental constitution) will be assessed. As well as age, gender, socioeconomic status, and type of gadget he or she is using frequently, and the purpose of gadget use will be assessed. After this period, participants will be randomly allocated to both intervention groups in a 1:1 allocation ratio. After this, Medhya Rasayana will be prescribed to participants in group I according to age-wise doses at a frequency of twice a day for a period of 90 days as mentioned in Table 1. Participants allocated to group II will be counseled regarding the adverse effects of gadget addiction on physical and mental health and advised to follow the following routine daily for a period of 90 days, details of nonpharmacological interventions are described in Tables 1 and 2. Yoga (sun salutations, meditation), Targeted reduction in Gadget use time Padanshik krama, that is, reduce gadget use time by one-fourth part each week. Reduced time will be used for adventure, bravery, cognitive skill-oriented things, sports, and yoga. Patients will be counseled to play indoor and outdoor games daily. Encouragement toward attending social programs whenever required and feasible, encouragement of parent-child relationship by advising parents to spend a minimum of 2 hours of quality time daily with children and to give a reward for not using mobile with encouraging children to learn new things, participate in group activities with peers and teach etiquette. Initial training in sun salutation and the method of meditation will be given to participants and their parents at the time of enrollment. Children will be asked to do the proposed sun salutation and meditation on the assessment day. Participants not responding or not following instructions will be eliminated from the study. Group III will be the external group due to the fact that cognitive behavioral therapy requires trained professionals and there are time and financial constraints as well. Hence, in this published data of previously conducted clinical trials, cognitive behavioral therapy intervention in the management of gadgets or internet addiction will be used for comparison with Medhya Rasayana and Ayurveda nonpharmacological interventions. On the 30th, 60th, and 90th days of treatment, participants will be evaluated using the internet addiction test scale and the *DSM-5* (*Diagnostic and Statistical Manual of Mental Disorders, Fifth Edition*) scale for social behavior. A follow-up will be conducted on the 180th day to determine whether the intervention’s possible effects are lasting.

**Inclusion and exclusion criteria.**

Inclusion criteria

- Participants irrespective of gender, religion, and socioeconomic status.
- Participants affected with gadget addiction as per case definition, that is internet addiction test score: 31-79.
- Age group between 6-16 years with signs and symptoms of gadget addictions as per *ICD-10* (*International Statistical Classification of Diseases, Tenth Revision*) or 11 as follows.
- Parents who are willing to provide written informed consent for their children to be included in the study.

Exclusion criteria

- Internet addiction test score >80.
- Children aged <6 years and those aged >16 years.
- Parents who are not willing to provide written informed consent for their children to be included in the study.
- Children experiencing from behavioral diseases, locomotor diseases, genetic diseases, and systemic disorders.

**Table 1:** Overview of interventions.

	Group P	Group N	Group C
Participants, n	80	80	External group
Name of medication	Medhya Rasayana	Nonpharmacological	Cognitive behavioral therapy
Dosage form	Young’s formula	As per schedule	—^a^
Duration	90 days	90 days	—
Assessment with IAT^b^ scale	0, 30, 60, and 90 days	0, 30, 60, and 90 days	—
Follow up with the IAT scale, digital addiction scale, and *DSM-5*^c^ scale	180th day	180th day	—
Route or mode of administration	Oral	—	—
Anupana	Lukewarm water	—	—

^a^Not applicable.

^b^IAT: internet addiction test.

^c^*DSM-5*: *Diagnostic and Statistical Manual of Mental Disorders* (Fifth Edition).

**Table 2:** Overview of Ayurveda nonpharmacological intervention.

Intervention	Details of method	Duration and frequency
Counseling (Satvavajaya)	Counseling about hazardous effects of mobile and benefits of mobile deaddiction	Fortnight 1 session with parents and child separately
Yoga therapy	Surya Namaskar, Dhyana	Daily
Encouragement for indoor and outdoor games	Patients will be counseled to play indoor and outdoor games daily	Daily for1hour
Encouragement towards attending social programs	Parents will be guided for engagement of children in learning new things, in group activities with peers, etiquette	“Si-opus sit” Whenever required and feasible
Encouragement of parent-child relationship	Parents are advised to spend time with their children. And advised to give a reward for not using mobile to child.	Minimum 2 hours of quality time daily by parents
Targeted reduction in Gadget use time	Reduced time will be used for adventure, bravery cognitive skill-oriented things, sports, and yoga.	By Padanshik karma reduces gadget use time by one-fourth part each week

### Participants

The required sample size was calculated by using the following formula.

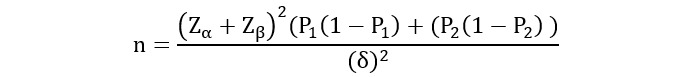

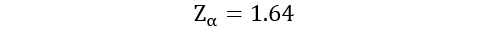

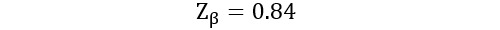







To evaluate the effectiveness of the therapeutic modalities for managing internet addiction, several key parameters are defined. The type I error rate () is set at 5% and the type II error rate (β) is set at 20%. The true proportion difference, represented by ε, is assumed to be 0. δ denotes the clinically relevant difference at 20% superiority. Initially, the proportion of individuals with internet addiction (P1) is 62.22% (0.622). After the intervention (P2), this proportion decreased to 36.2% (0.362). To determine the appropriate sample size for the study, a clinically accepted difference of 20% is used. The sample size is calculated according to the formula N=(1.64+0.84)2(0.622)(1–0.622) +0.362(1–0.362)/(0.20)2. Based on this formula, the required sample size is 72 individuals per group. To account for a 10% dropout rate, the sample size is increased to 80 per group.

Therefore, 80 participants will be recruited after randomization in each group from the Kaumarbhritya OPD of Mahatma Gandhi Ayurved College Hospital and specialty camps held in peripheral villages of Wardha city. A computer-generated random number table will be used to avoid selection bias in the study. The principal investigator will carry out the process of randomization and enroll the participants. To be eligible for participation, participants should fulfill the inclusion criteria as mentioned in Textbox 2. Patients having any characteristics of exclusion criteria will be excluded from participation in the study.

### Preparation of Medhya Avaleha

Medhya Rasayana Avaleha is the polyherbal combination of Centella asiatica, Convolvulus prostratus, Tinospora cordifolia, and Glycyrrhiza glabra mentioned in Table 3. Drugs required for the preparation of Medhya Avaleha will be collected from local markets or herbal gardens and identified in the Dravyaguna Department. Preparation of Medhya Rasayana Avaleha will be done as per classical reference [25] in Rasashastra and Bhaishyajya Kalpana Department of MGACHRC, Salod Wardha.

**Table 3:** Overview of contents of Medhya Rasayana Avaleha [26].

Contents	Parts used	Proportion	Maximum dose
Centella asiatica (Linn.); family: Apiaceae; Ayurveda name: Mandukparni	Leaves	1 part	mg/kg [27]
Convolvulus prostratus (chois); family: Convolvulaceae; Ayurveda name: Shankhpushpi	Leaves, roots, barks, flowers,	1 part	mg/kg [28]
Tinospora cordifolia (Willd Miers); family: Menispermaceae; Ayurveda name: Guduchi	Barks	1 part	Extracts 120 mg/kg [29]
Glycyrrhizaglabra (Linn.); family: Fabaceae; Ayurveda name: Yashtimadhu	Roots	1 part	50 gm/day [30]

### Yoga Pose

**Figure 2:**
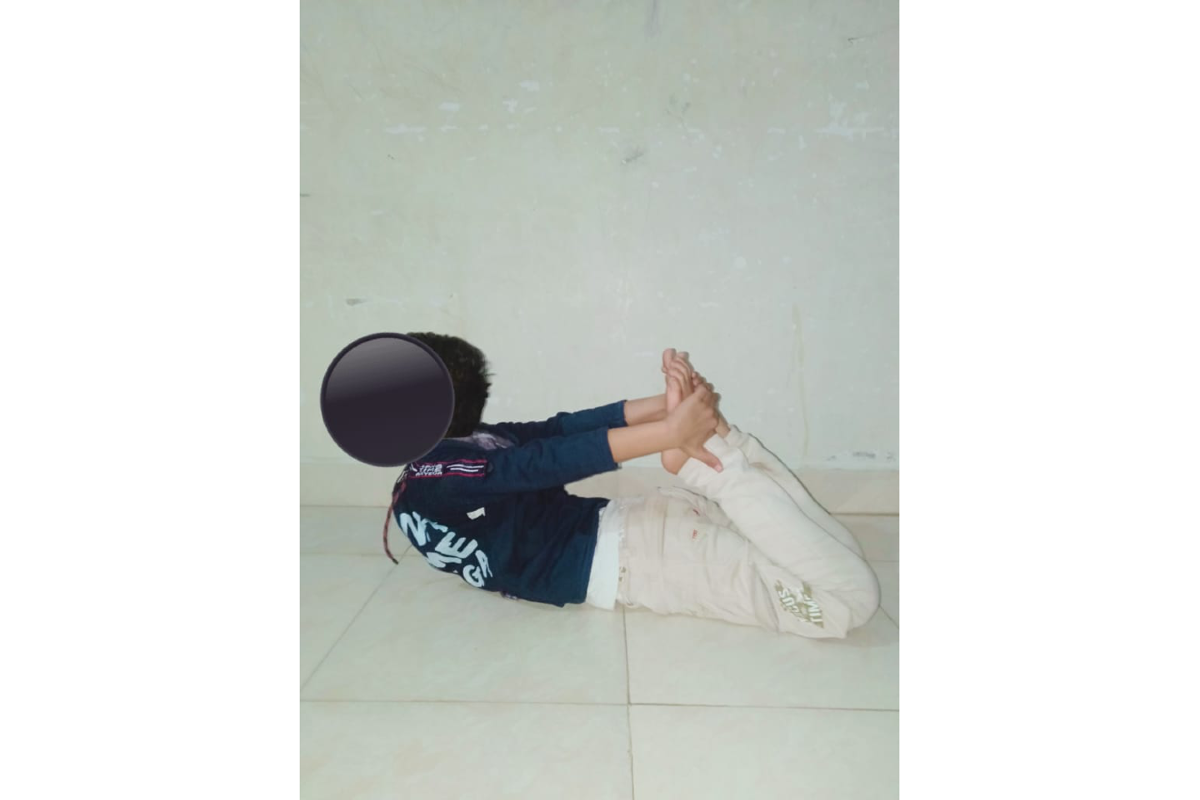
Yoga pose 1.

**Figure 3:**
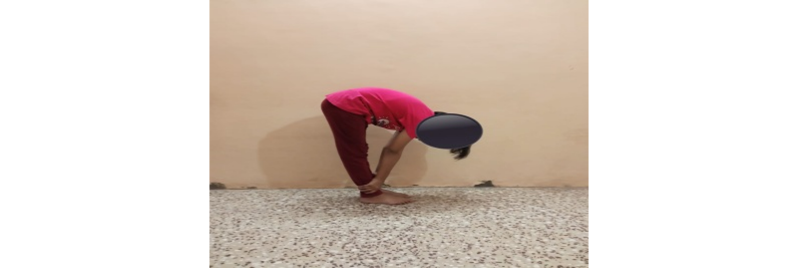
Yoga pose 2.

**Figure 4:**
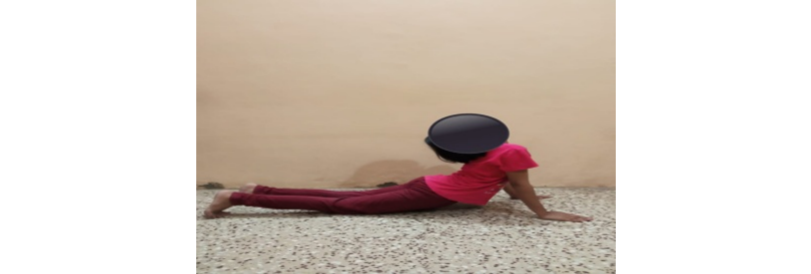
Yoga pose 3.

For yoga poses refer to Figures 2-4.

### Assessment of Participants

All participants will be assessed for level of addiction, social behavior, and Prakruti (physical and mental constitution) before initiating the intervention during the study at 0, 30, 60, and 90 days, and on the follow-up day at180 days at Mahatma Gandhi Ayurved Hospital Salod (H). For the assessment of gadget addiction, an internet addiction test scale [31] questionnaire and a digital addiction scale [32] will be used. There are 20 statements on this questionnaire. After carefully reading each statement, participants have to choose the response (0, 1, 2, 3, 4, or 5) that best describes him or her using the 5-point Likert scale. Select the option that most accurately reflects how participants have been spending their time over the past month if 2 options seem to fit equally well. Along with participants, their parents or caretakers will be asked the following question regarding the gadget-addicted behavior of their children and the mean of both scores will be calculated to avoid bias in the study. The social behavior of the patient will be assessed by *DSM-5* [33] criteria on the proposed day Prakruti examination will conducted on the day of initiation of intervention by Ayusoft software [34].

### Data Quality Assessment

The researchers will oversee and direct the process of gathering data. Since this study is a component of a postgraduate doctoral degree project, no committee was specifically tasked with overseeing or monitoring the data trials. However, the study progress will be discussed monthly with the supervisor.

#### Statistical Analysis

The data collection phase will be concluded with statistical analysis. Repeated measures ANOVA test, and chi-square test, will be used for statistical interpretation. A statistician consultation will be considered for the selection of the proper statistical method.

#### Dropout

If participants choose to stop the intervention, a detailed interview of the participant will be conducted to rule out the reason for the dropout. But no force will be exerted on them to continue the intervention. Common reasons for dropping out of the study will be enlisted and measures will be taken to manage them if feasible within the limits of the researcher.

#### Informed Consent

Before the study begins, all participants must give written informed consent (ie, during the first visit to the hospital). Every participant will get information regarding the study’s design, objectives, data confidentiality, and the freedom to withdraw from the study at any moment without giving a reason. The original informed consent or approval from the institutional review board explicitly permits secondary analyses of the research data without necessitating additional consent.

#### Adverse Effects

Adverse consequences are characterized as unfavorable results resulting from study involvement. This study’s potential side effects may include injury while doing yoga or Gastrointestinal system disturbances due to Medhya Rasayana avaleha. Adverse effects that occur will be noted and assessed for both the intervention and control groups.

#### Data Storage

Every piece of information regarding the study will be stored on a central disk space on a password-protected computer. Data access will be restricted to members of the research team only.

#### Incentive

Participation in the study is completely voluntary; hence, no incentive will be provided to students.

### Ethical Considerations

The Mahatma Gandhi Ayurved College Hospital and Research Center’s (MGACHRC) Institutional Ethics Committee approved this study (registration number MGACHRC/IEC/Oct-2022/614), and it was also registered with the Clinical Trial Registry of India (registration number CTRI/2021/10/037211). We will get assent from each participant’s parents. During data analysis, measures will be taken to protect participants’ privacy. The original informed assent or approval from the institutional review board explicitly permits secondary analyses of the research data without necessitating additional assent.

## Results

In December 2023, the randomized controlled study got underway. Since participants may be in the study at any time, our goal is to complete the research by December 2024. Any significant changes to the protocol will be posted on clinicaltrials.gov. The study’s findings will be disseminated through publications.

## Discussion

### Principal Findings

Compulsive conduct is one of the symptoms of addiction that can substantially impede a person’s ability to receive therapy. Gadgets negatively affect the social life, health, and cognition of the child [35]. Hence, it is necessary to diagnose and treat gadget addiction in children as early as possible. The Medhya Rasayana avaleha was selected in this study as a pharmacological intervention. Medhya Rasayana has been popularly used as a proven therapy for the improvement of the intellect of children in India for centuries. As per Ayurveda, cognition is called Medha and it is not only related to the memory of a person but also consists of 3 components, that are Dhee (intellect), Dhruti (control of mind), and Smriti (memory). Among these 3 components, Dhruti is responsible for governing features and prevents the person from consuming destructive or nonbeneficial substances [36]. Consumption of Medhya Rasayana on a daily basis could enhance the control of the mind along with intellect and memory, which is beneficial in the management of addiction.

The nonpharmacological arm selected in this study is Satvavajaya (Ayurveda psychotherapy). It is the psycho-spiritual method and is intended for use with the mind and its associated qualities. It is mainly used as an important therapeutic to control the mind, which is heading toward unwholesome objects. Satvavajaya has multiple domains along with them, we are choosing the Vijnana (educative approach), Dhairya (supportive approach and development of coping ability), and Samadhi (psychophilosophical approach) [37]. Vijana (educative approach) and Dhairya (supportive approach and development of coping ability) are achieved in this treatment through proper counseling, which will include the adverse effects of gadget addiction on physical and mental health in a simple language that the child can understand. As well, Padanshika karma is preferred over sudden stoppage of treatment, which is part of Dhairya (supportive approach and development of coping ability). Samadhi (psychophilosophical approach) is a stage of unwavering concentration and minimizing mental fluctuations one could be distracted from unwholesome things and thoughts. This is achieved by regular meditation and yogic practices. Child externalization behavior and psychological factors like anxiety and depression are associated with gadget addiction [38,39]. The Ayurveda interventions, Medhya Rasayana and Satvavajaya have proven therapeutic effects on psychological factors [40-46]. As well as the impaired cognition in gadget addiction [47], both pharmacological and nonpharmacological interventions are beneficial in improving cognition [48].

Gadget addiction in children is one of the serious issues of concern in today’s era so, there is a need to deaddict children to maintain proper growth and development of the child. Gadget addiction in children is associated with many behavioral diseases and no cost-effective convenient therapy is available for it this study can fill the gap.

While conducting the study, the investigator may face difficulties at the level of overdiagnosis of gadget addiction in children due to overconscious parents. Another difficulty we may face in the Ayurveda nonpharmacological intervention group is the failure to follow lifestyle modifications and yogic practices. This is the first study in the Ayurveda domain using pharmacological and nonpharmacological interventions for gadget addiction. Hence the small sample size and lack of a proven Ayurvedic control group are the main limitations of this study.

### Conclusion

This research will provide crucial new information about the relative effectiveness of Ayurveda nonpharmacological therapies and Medhya Rasayana in treating children’s gadget addiction. The results will guide evidence-based treatments aimed at reducing the negative impact of excessive gadget use on this susceptible population’s psychosocial development. In the end, the findings are meant to help policy makers and medical professionals create sensible plans to deal with the rising issue of childhood gadget addiction.

## Abbreviations

C-DAC: Centre for Development of Advanced Computing
*DSM-5*: *Diagnostic and Statistical Manual of Mental Disorders* (Fifth Edition)
MGACHRC: Mahatma Gandhi Ayurved College Hospital and Research Center

## Data Availability

The datasets that will be generated and/or analyzed during this study will be available from the corresponding author upon reasonable request.
